# Chiral Nanoparticles Force Neural Stem Cell Differentiation to Alleviate Alzheimer's Disease

**DOI:** 10.1002/advs.202202475

**Published:** 2022-08-25

**Authors:** Baimei Shi, Jing Zhao, Zhuojia Xu, Chen Chen, Liguang Xu, Chuanlai Xu, Maozhong Sun, Hua Kuang

**Affiliations:** ^1^ International Joint Research Laboratory for Biointerface and Biodetection Jiangnan University Wuxi Jiangsu 214122 China; ^2^ State Key Laboratory of Food Science and Technology Jiangnan University Wuxi Jiangsu 214122 China

**Keywords:** Alzheimer's disease, chiral, gold nanoparticles, light, neural stem cells

## Abstract

The differentiation of neural stem cells via nanomaterials has attracted attention and has become a potential tool. However, the chirality effect in neural stem cell differentiation has not been investigated. Here, this study shows that chiral nanoparticles (NPs) with strong chirality can efficiently accelerate the differentiation of mouse neural stem cells (NSCs) into neurons under near‐infrared (NIR) light illumination. L‐type NPs are 1.95 times greater than D‐type NPs in promoting NSCs differentiation due to their 1.47‐fold endocytosis efficiency. Whole gene expression map analysis reveals that circularly polarized light illumination and chiral NPs irradiation significantly upregulate *Map2*, *Yap1*, and *Taz* genes, resulting in mechanical force, cytoskeleton protein action, and accelerated NSCs differentiation. In vivo experiments show that successful differentiation can further alleviate symptoms in Alzheimer's disease mice. Moreover, the clearance of L‐type NPs on amyloid and hyperphosphorylated p‐tau protein reachs 68.24% and 66.43%, respectively, under the synergy of NIR irradiation. The findings suggest that strong chiral nanomaterials may have advantages in guiding cell development and can be used in biomedicine.

## Graphical abstract

The optical activity generated by strong chiral nanoparticle under linear polarized light illumination has significantly alleviated the pathological behavior of AD mice. The developed strategies make in‐depth research possible to open up an avenue for treatment of neurodegenerative diseases and tissue regeneration via remote chiral photon control.

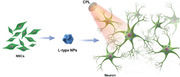

## 1 Introduction

Alzheimer's disease (AD) is considered one of the most deadly and burdensome diseases of this century.^[^ ^1^ ^]^ The most typical pathological features of AD are extracellular deposition of *β*‐amyloid (A*β*) proteins, p‐tau neurofibrillary tangles, and local inflammation, which gradually lead to loss of the connection between neurons and synapses, resulting in neuronal loss.^[^ ^2^, ^3^, ^4^ ^]^ There are currently no permanent remedies for AD, and the main treatment modalities are divided into two broad categories, nonpharmacological and pharmacological.^[^ ^1^, ^5^ ^]^ Nonpharmacological, multidisciplinary interventions based primarily on lifestyle modification to prevent cognitive decline include moderate physical activity, healthy nutrition, cognitive training, and management of vascular and metabolic risks.^[^ ^6^ ^]^ Pharmacological therapeutic drugs are mainly divided into three types. The first includes drugs that enhance cognitive status, including cholinesterase inhibitors and memantine.^[^ ^7^, ^8^ ^]^ These are followed by drugs for psychiatric symptoms caused by AD, Pimavanserin, a 5‐HT_2A_ receptor reverse agonist, has been submitted to the U.S. Food and Drug Administration as a treatment for dementia‐related psychosis.^[^ ^9^ ^]^ Recent studies have also supported the treatment of AD‐induced agitation symptoms with citalopram (a selective serotonin reuptake inhibitor) and brexpiprazole (an atypical antipsychotic).^[^ ^10^ ^]^ Finally, there are drugs that improve the disease itself. Some of these drug treatments are in the advanced stages of clinical trials, including anti‐A*β* protein, anti‐tau, and anti‐inflammatory strategies, such as BAN2401, Aducanumab, and gantenerumab.^[^ ^11^, ^12^, ^13^ ^]^ However, the drugs currently used in AD treatment have certain drawbacks, such as low bioavailability, blood–brain barrier penetration, and low half‐life. In addition, these approaches ignore the loss of neurons in damaged tissue, slowing disease control or treatment.

Neural stem cells (NSCs) have pluripotent and significant regenerative potential, and are proposed for the treatment of neurodegenerative diseases as well as stroke and spinal cord injury.^[^ ^14^, ^15^, ^16^ ^]^ The fusion of stem cell therapies and nanoparticles (NPs) offers great promise in the study, diagnosis and treatment of neurodegenerative diseases.^[^ ^17^, ^18^, ^19^, ^20^ ^]^ A growing number of preclinical studies suggest that the transfer of NSCs could lead to potential new treatments for neurodegenerative diseases.^[^ ^21^, ^22^ ^]^

Chiral NPs with specific optical signal responses are widely used as biological probes for highly sensitive detection of biomolecules, catalysis, and biosensors.^[^ ^23^, ^24^, ^25^, ^26^, ^27^, ^28^, ^29^, ^30^ ^]^ In addition, chiral NPs have better biocompatibility, can effectively reduce the toxic effect on cells, and have higher safety when interacting with biomolecules.^[^ ^24^, ^31^, ^32^, ^33^, ^34^ ^]^ They can also enhance the uptake capacity of cells, have stronger sustained release ability, and specific enantiomer selectivity, which can improve the therapeutic effect in tumors, neurodegenerative diseases, and other diseases.^[^ ^35^, ^36^, ^37^, ^38^ ^]^ It was found that ultrasmall chiral gold NPs (3.3 nm) can treat AD by inhibiting A*β* protein aggregation.^[^ ^36^ ^]^ However, there are few studies on the interaction between chiral NPs and stem cells, and on the treatment of AD by promoting the growth and differentiation of NSCs.

**Scheme 1:**
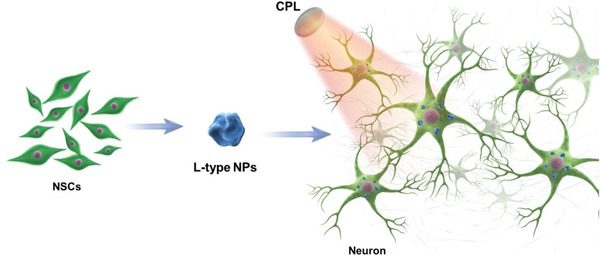
Illustration of neural stem cell differentiation pathway in AD mice induced by chiral NPs under 808 nm NIR laser irradiation.

In the present study, the effects of chiral NPs with high *g*‐factor values on mouse NSCs differentiation were investigated under 808‐nm circularly polarized light (CPL) illumination (**Scheme** **1**). It was found that cell differentiation efficiency increased as strong chiral NPs. Finally, chiral NPs were injected into the brain of AD model mice by stereotactic in situ injection, and the mice were treated for 90 d under CPL illumination. It was found that the strong chiral NPs had obvious therapeutic effect.

## 2 Results and Discussion

### 2.1 Effect of Chiral NPs on NSCs Differentiation under CPL

Chiral NPs were synthesized according to a previous report,^[^ ^39^ ^]^ and the nanoprism was also used as a control. A 808 nm laser was selected to examine the effect of chiral NPs on the differentiation of NSCs under CPL illumination. For the follow‐up experiments, the irradiation time and irradiation power of chiral NPs were optimized, and the following optimal conditions for chiral NPs to prevent the generation of heat after NIR irradiation were selected: 200 mW cm^−2^ and 5 min (Figures S1 and S2, Supporting Information). In order to evaluate the potential biological application of chiral NPs, their biocompatibility was investigated by cell viability assay. The nanoprism, L‐type NPs, and D‐type NPs were incubated with NSCs for 24 h, and cell viability was determined using the Cell Counting Kit‐8 (CCK‐8). When the concentrations of different chiral NPs reached 20 × 10^−9^ m, cell viability was basically unchanged, and the NPs were applied in subsequent experiments (Figure S3, Supporting Information).

The amount of intracellular chiral NPs after incubation with NSCs was qualified by two‐photon luminescence (TPL) intensity (Figures S4 and S5, Supporting Information). This indicated that L‐type NPs had higher endocytosis efficiency, which was 1.47 times that of D‐type NPs and 1.82 times that of the nanoprism.

The effect of different chiral NPs on NSCs differentiation was then investigated under CPL irradiation. NSCs incubated with L‐type NPs were illuminated under left circularly polarized (LCP) light for 7 d (200 mW cm^−2^, 5 min d^−1^), and NSCs incubated with D‐type NPs were illuminated under right circularly polarized (RCP) light, or with nanoprism under linearly polarized (LP) light.

**Figure 1:**
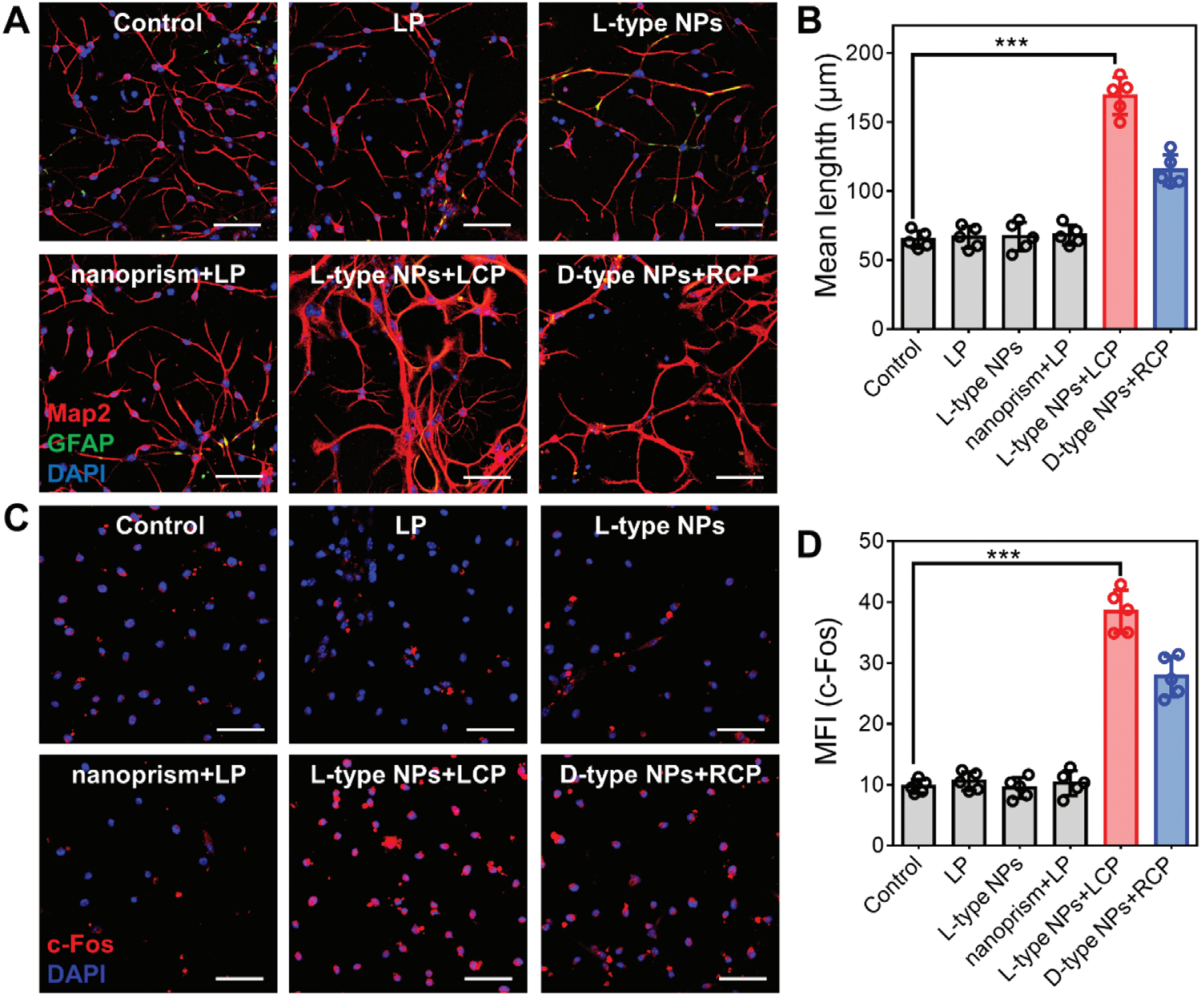
A) Confocal images of NSCs incubated for 7 d under different experimental conditions (Control, LP light only, L‐type NPs only, nanoprism under LP light, L‐type NPs under LCP light, and D‐type NPs under RCP light). In the group containing the material, the material was incubated with NSC for 12 h every day, the medium was replaced and then irradiated with CPL (200 mW cm^−1^,^[^ ^2^ ^]^ 5 min), incubated for another 12 h, and the new medium and materials were replaced. Cells without nanoparticles or light exposure were used as control. Red: Map2 for mature neurons. Blue: DAPI for the nucleus. Green: GFAP for astrocytes. Scale bar, 100 µm. B) Mean lengths of neurites in differentiated NSCs (A). C) Confocal images of c‐Fos protein in NSCs incubated with different experimental conditions. Red: c‐Fos for neurons activation. Blue: DAPI for the nucleus. Scale bar, 100 µm. D) Mean fluorescence intensity (MFI) in (C). ***p < 0.001. Data are presented as the mean ± s.d. (*n* = 5).

From **Figure** **1**A,B and Figures S6 and S7 (Supporting Information), it can be seen that with the extension of growth time, NSC neurite length in each group gradually increased. The growth of cells in the light only group, L‐type NPs only group, and nanoprism group was similar to that in the control group. However, NSC neurite length increased after incubation with L‐type NPs or D‐type NPs under CPL illumination for 7 d, respectively. The NSC neurite length in the L‐type NPs and D‐type NPs group changed significantly, and were 2.67 times (173.5 µm) and 1.76 times (114.21 µm) greater than that in the control group (64.8 µm), respectively.

**Figure 2:**
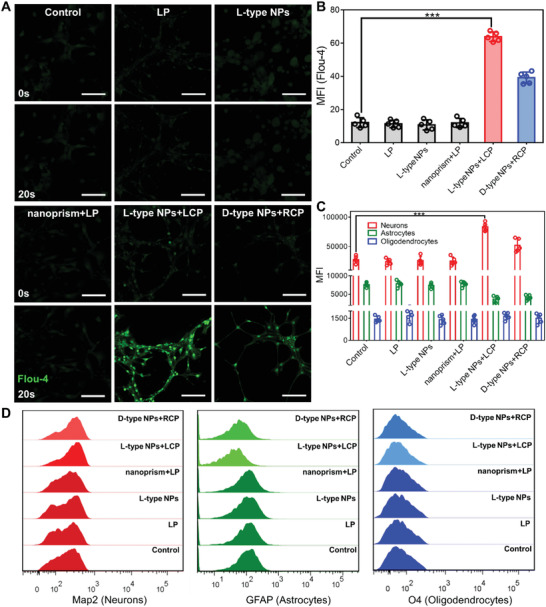
A) Ca^2+^ image of NSCs cultured with different experimental conditions after addition of 10 × 10^−9^ m carb (agonist). Scale bar, 100 µm. B) Mean fluorescence intensity (MFI) in (A). C) Mean fluorescence intensity (MFI) in (D). D) Neurons, astrocytes, and oligodendrocyte detection by flow cytometry in differentiated NSCs with different experimental conditions (Alexa Fluor 555 labeled Map2; Alexa Fluor 488 labeled GFAP; Alexa Fluor 633 labeled O4). ***p < 0.001. Data are presented as the mean ± s.d. (*n* = 5).

The c‐Fos protein is considered a regulator of cell proliferation, differentiation, and transformation, as well as a marker of neuronal activity.^[^ ^40^ ^]^ From Figure 1C,D it can be seen that c‐Fos protein content significantly increased in the group incubated with L‐type NPs or D‐type NPs under CPL illumination. The content of c‐Fos protein in the L‐type NPs group was 2.29 times that in the D‐type NPs group, indicating that chiral NPs increased c‐Fos in neurons under CPL illumination. The effect of L‐type NPs was markedly better than that of D‐type NPs. The differentiation of NSCs was accelerated by L‐type NPs or D‐type NPs under CPL illumination, demonstrating the biological function of neurons. Rapid calcium channel activity was observed on confocal microscopy images (**Figure** **2**A,B). By contrast, cells not incubated with chiral NPs, showed no characteristic changes in calcium ion levels after illumination.

The differences in NSCs differentiation under these light conditions were further confirmed by flow cytometry (Figure 2C,D). The fluorescence intensity of neurons in the group containing chiral NPs was ≈5–20 times higher than that of astrocytes. The difference in the proportion of differentiation was greatest in the group containing L‐type NPs, and the fluorescence intensity of differentiation in neurons was 25.02 ± 5.42 times that in astrocytes, which was significantly higher than that in the D‐type NPs group (12.83 ± 3.12 times) and nanoprism group (3.22 ± 0.71 times). Changes in oligodendrocytes were not obvious, accounting for 3.48% ± 0.88% of the total cells in each group. These results showed that chiral NPs promoted NSCs differentiation under CPL illumination and the effect of L‐type NPs was significantly better than that of D‐type NPs. Experiments carried out under the same concentration of intracellular chiral NPs and the same conditions showed consistent results with those mentioned above (Figures S8–S12, Supporting Information). Chiral NPs promoted increased differentiation of NSCs under CPL illumination.

### 2.2 Mechanism of Chiral NPs on NSCs Differentiation

**Figure 3:**
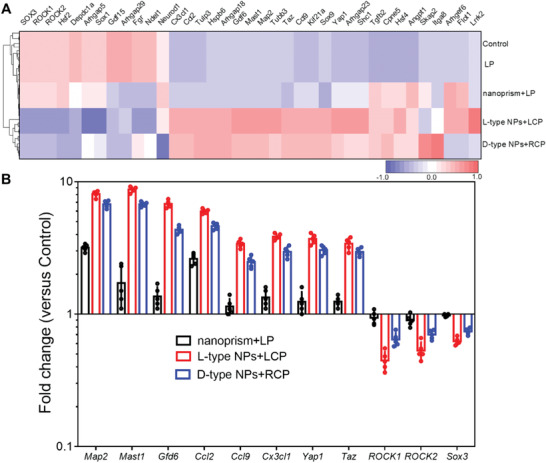
A) Global gene‐expression pattern of transcription factors in NSCs incubated with different experimental conditions for 7 d. B) The differentially expressed genes from the heatmap results are expressed as fold change compared with levels in Control (three technical pools of 1 × 10^6^ cells were averaged per biological replicate).

The overall gene expression profile of the effect of L‐type NPs and D‐type NPs on NSCs under CPL illumination was studied (**Figure** **3**A). Compared with the control group, the degree of activation of NSCs genes by different chiral NPs was notably different, and the more obvious changes were *Map2*, *Mast1*, *Gdf6*, *Ccl2*, *Ccl9*, *Cx3cl1*, *Yap1*, *Taz*, *ROCK1*, *ROCK2*, and *Sox3*. Of these, genes *Map2*, *Mast1*, *Gdf6*, *Ccl2*, *Ccl9*, *Cx3cl1*, *Yap1*, and *Taz* were upregulated; and *ROCK1*, *ROCK2*, and *Sox3* were downregulated (Figure 3B).

The upregulation of *Map2* was positively correlated with the differentiation of NSCs into neurons. Under CPL illumination, the upregulation intensity of *Map2* gene was increased 8.082 times compared with the control group after L‐type NPs treatment, which was higher than that in the D‐type NPs group (6.76 times) and nanoprism group (3.18 times). The downregulated expression of *ROCK2* and *ROCK1* was negatively correlated with axon growth.^[^ ^41^ ^]^ Under CPL illumination, L‐type NPs had a significant effect on *ROCK2* and *ROCK1* genes after NSCs reacted with the nanoprism, L‐type NPs and D‐type NPs. These results indicated that under CPL, the promoting effect of L‐type NPs on NSCs differentiation was greater than that of D‐type NPs.

The upregulation of *Mast1* was positively correlated with tubulin and the cytoskeleton. The changes in *Gdf6* were related to cell proliferation and neuronal cell protection, and upregulation of *Gdf6* enhanced the protection of neuronal cells.^[^ ^42^ ^]^ In addition, the upregulation of *Ccl2*, *Ccl9*, and *Cx3cl1* promoted the differentiation of NSCs.^[^ ^43^ ^]^ Similarly, when the control group was set as a reference, it was found that the regulation of L‐type NPs on *Mast1* and *Gfd6* was much higher than that of the nano‐prism, and was 5.08 and 4.91 times, respectively. In addition, under the same *g*‐factor value, L‐type NPs regulated genes better than D‐type NPs, and the upregulated levels of *Ccl2*, *Ccl9*, and *Cx3cl1* were 1.29, 1.37, and 1.30 times higher than those with D‐type NPs, respectively.

*Yap1* and *Taz* genes have been demonstrated to be mechanical sensors, sensing and transducing mechanical signals at the molecular level to regulate cell growth shape and extracellular matrix stiffness.^[^ ^44^ ^]^ Under LCP illumination, the effect of L‐type NPs on the *Yap1* and *Taz* genes in NSCs was 2.98 and 2.73 times that of the nanoprism under LP illumination, respectively. The effect of D‐type NPs on these two genes in NSCs under RCP illumination was lower than that of L‐type NPs (1.55‐fold lower for *Yap1*; 1.31‐fold lower for *Taz*).

Previous studies have proved that the cytoskeleton and nucleus of NSCs can receive periodic forces from CPL illumination, thereby accelerating the differentiation of NSCs.^[^ ^45^ ^]^ In our experiments, we found that under light alone, cells were genetically similar to controls (Figure 3A). However, under CPL illumination, chiral NPs showed significant changes in mechanotransduction signals, indicating that only the simultaneous action of chiral NPs and light promoted cell differentiation. According to Figures S4 and S5 (Supporting Information), cells had higher endocytosis efficiency when incubated with L‐type NPs; thus, L‐type NPs promoted greater cell differentiation. *Sox3* was expressed by relatively static progenitor cells in the forebrain of newborns, and progenitor cells proliferate actively with decreased cell differentiation as they age.^[^ ^46^ ^]^ In addition, L‐type NPs had the most obvious effect on *Sox3* after interacting with NSCs under LCP illumination. Therefore, chiral NPs pronounced the regulation of genes under CPL illumination. Moreover, L‐type NPs regulate genes better than D‐type NPs.

### 2.3 Application of Chiral NPs in AD Mice

**Figure 4:**
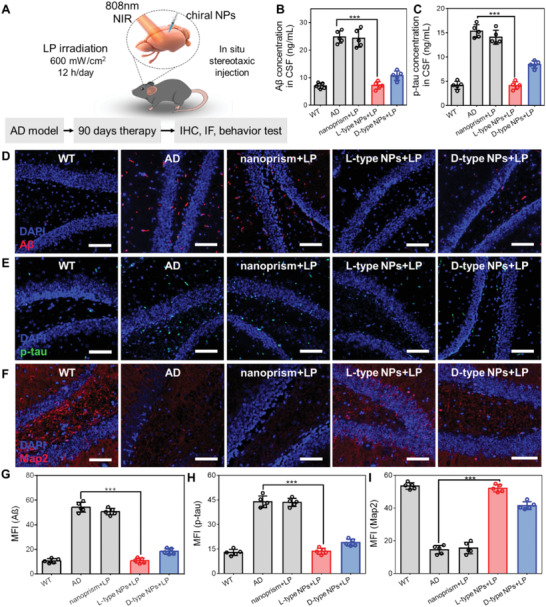
A) Schematic representation of AD model mice treated by different treatment conditions (Normal saline injection was used as WT group; LP irradiation group only; L‐type NPs only; nanoprism, L‐type NPs or D‐type NPs under LP irradiation. In the added LP light group, the light was 12 h a day, intensity 600 mW cm^−2^). B,C) A*β* and p‐tau concentration in cerebrospinal fluid of AD mice after different treatment conditions. D–F) Immunofluorescence of A*β*, p‐tau, and Map2 in the hippocampal of AD mice after different treatments. Scale bars, 50 µm. G–I) Mean fluorescence intensity of A*β*, p‐tau, and Map2 in the hippocampal after different treatments. (*n* = 5 mice per group). ***p < 0.001. Data are presented as the mean ± s.d. (*n* = 5).

The AD model mice (2xTg‐AD) with PrP‐HAPP/hPS1 double transgenic technology were selected to determine the biological function of different chiral NPs in promoting the differentiation of NSCs into neurons in mouse brain under LP illumination. Chiral NPs (5 mg kg^−1^) were injected into the brains of AD mice by in situ stereotaxic injection, respectively (anteroposterior, −1.7 mm; mediolateral, ± 1.5 mm; dorsoventral, −1.5 mm),^[^ ^45^ ^]^ and were irradiated with 808 nm NIR light illumination (irradiation power: 600 mW cm^−2^) for 12 h d^−1^, and continuous irradiation for 90 d (**Figure** **4**A). Hematoxylin and eosin (H&E) staining of the heart, liver, spleen, lungs, and kidneys of each group of mice showed no significant abnormalities, indicating that chiral NPs did not have a toxic effect on mice (Figure S13, Supporting Information).

The A*β* and hyperphosphorylated tau (p‐tau) proteins were extracted from the cerebrospinal fluid (CSF) of AD mice in each group, these two proteins are markers of AD, and the protein content was determined using an ELISA kit. It was found that chiral NPs reduced the content of A*β* and p‐tau proteins under 808 nm NIR light irradiation (Figure 4B,C and Figure S14, Supporting Information). L‐type NPs had the best therapeutic effect under 808 nm NIR illumination. After 90 d of treatment, the concentrations of A*β* and p‐tau proteins were 7.19 ± 1.18 and 4.12 ± 0.69 ng mL^−1^, respectively, and were close to the levels observed in the wild‐type (WT) group. The concentration of A*β* and p‐tau proteins in AD mice treated with light alone, L‐type NPs alone, or nanoprism with NIR light was similar to that in AD mice treated with PBS alone, and was maintained at around 24.99 ± 1.29 and 13.69 ± 1.56 ng mL^−1^, respectively.

**Figure 5:**
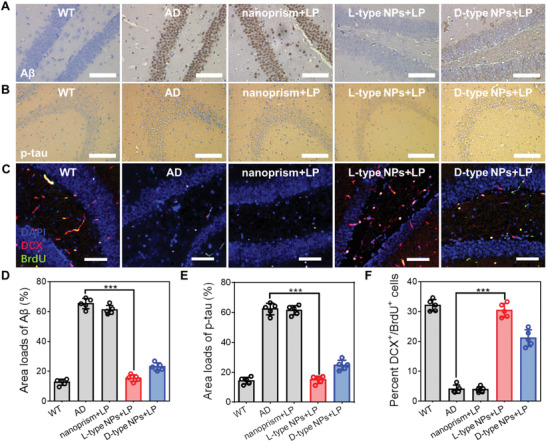
A,B) Representative immunostaining of hippocampal sections for A*β* and p‐tau protein aggregates of the WT mice and AD mice after different treatment conditions. Scale bars, 50 µm. C) Representative hippocampal section images of the WT mice and AD mice with different treatments, immunostained for DCX (red) to label immature neurons, BrdU (green) to label dividing cells, and DAPI (blue) to stain nuclei. Scale bars, 50 µm. D) Quantitative analysis of A*β* loads in (A). E) Quantitative analysis of p‐tau loads in (B). F) Quantification of the overall fraction of newborn hippocampal cells in (C) that underwent neuronal differentiation using stereological estimation. (*n* = 5 mice per group). ***p < 0.001. Data are presented as the mean ± s.d. (*n* = 5).

The above results were further confirmed by immunofluorescence and immunohistochemistry in the mouse hippocampus. The content of A*β* and p‐tau proteins in the L‐type NPs with NIR light group was significantly reduced, and the content of Map2 was also close to that in the WT group (Figures 4D–I and **5**A,B,D,E, and Figures S15–S19, Supporting Information). Immunofluorescence staining of the hippocampal area of mice in the L‐type NPs group under NIR illumination showed that after 90 d of treatment, the fluorescence intensity of A*β* protein decreased by 79.21% compared with that in the AD group, and the fluorescence intensity of p‐tau protein decreased by 68.89%. The fluorescence intensity of Map2 was 49.83%, which was similar to that in the WT group (53.53%). Immunohistochemical results also showed that the positive proportion of A*β* protein in the hippocampus of the L‐type NPs group decreased from 65.35% to 15.42% compared with the AD group, and the positive proportion of p‐tau protein decreased from 62.20% to 14.93%.

**Figure 6:**
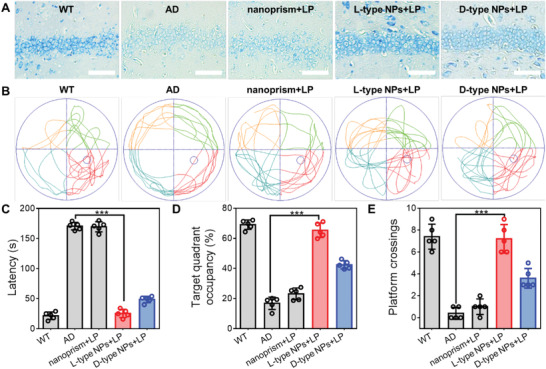
A) Nissl staining of neuro cells in the brains (hippocampus) of the WT mice and AD mice with different treatments. B) The track sheets of the WT mice and AD mice after different treatments. C) The latent period in water maze of the WT mice and AD mice to find the target quadrant after different treatments. D) The time in the target quadrant of the WT mice and AD mice after different treatments. E) The crosses times in target quadrant of the WT mice and AD mice after different treatments. (*n* = 5 mice per group). ***p < 0.001. Data are presented as the mean ± s.d. (*n* = 5).

Doublecortin (DCX) is a marker of newborn neurons, and bromodeoxyuridine (BrdU) is a marker of proliferating cells.^[^ ^47^ ^]^ The ratio of the two represents the proportion of new neurons in proliferating cells. From Figure 5C,F and Figure S20 (Supporting Information), it can be seen that chiral NPs increased the proportion of DCX in BrdU‐positive cells under 808 nm NIR illumination. The number of new neurons in L‐type NPs was twice that in D‐type NPs. These findings indicated that chiral NPs promote differentiation of NSCs into neurons in the hippocampus under 808 nm NIR illumination, increasing the proportion of new neurons. The most obvious effect under 808 nm NIR illumination was seen in the L‐type NPs group. Nissl staining also revealed that the neuronal nuclei in the L‐type NPs group were intact and increased in number under 808 nm NIR illumination compared with the PBS group (**Figure** **6**A and Figure S21, Supporting Information).

In order to evaluate the therapeutic effect of different treatment methods on AD model mice after 90 d of treatment, the Morris water maze (MWM) test was used to evaluate the spatial cognitive memory ability of model mice (Figure 6B–E and Figure S22, Supporting Information). The mice in each group were trained to find the platform in the pool over 5 d, and the platform was removed for testing on the sixth day. Compared with mice in the AD group, the AD model mice treated with different chiral NPs under NIR light illumination showed significantly better spatial learning and memory. The AD mice with L‐type NPs under 808 nm NIR illumination remained in the target quadrant for ≈61.28% of the total time, and the number of times they crossed the platform also increased to approximately seven times as compared with the AD mice group. By contrast, AD mice without treatment showed significant deficits, and when the platform was removed, the mice reached and remained in the target quadrant for a short time, only about 7.6% of the total time.

These data suggest that chiral NPs reduced the content of A*β* and p‐tau, and promoted the proportion of neuronal differentiation in AD mice, which had obvious therapeutic effect. The L‐type NPs group effectively reduced the content of A*β* and p‐tau in AD mouse brains, and gradually restored memory function in AD mice under 808 nm NIR illumination.

## 3 Conclusion

In summary, chiral NPs with strong optical activity accelerated the differentiation of NSCs into functional neurons when irradiated with CPL. In situ injection of strong chiral NPs into mouse brain significantly reduced the content of A*β* and p‐tau protein in AD mice under LP irradiation, and restored the pathological behavior of AD mice. Based on the effectiveness of directed differentiation of NSCs, we hypothesize that the optical activity generated by strong chiral NPs under LP illumination may facilitate the development of cell culture and cell engineering, especially in stem cell therapy. Meanwhile, according to the favorable results of this therapeutic AD model, the synergistic effect of this strong chiral material in AD therapeutic strategies makes in‐depth research possible to provide opportunities for the application of chiral materials in the treatment of neurodegenerative diseases and tissue regeneration.

## Conflict of Interest

The authors declare no conflict of interest.

## Supporting information

Supporting Information

## Data Availability

The data that support the findings of this study are available in the Supporting Information of this article.
