# Supplementary material for: Chiral Nanoparticles Force Neural Stem Cell Differentiation to Alleviate Alzheimer's Disease
Source: Adv Sci (Weinh). 2022 Aug 25;9(29):2202475. doi: 10.1002/advs.202202475 (PMC9561871; doi:10.1002/advs.202202475)
Supplement: Supplementary file 1 — Supporting Information [file ADVS-9-2202475-s001.pdf]

ADVANCED SCIENCE
Open Access

Supporting Information

for *Adv. Sci.*, DOI 10.1002/advs.202202475

Chiral Nanoparticles Force Neural Stem Cell Differentiation to Alleviate Alzheimer's Disease

Baimei Shi, Jing Zhao, Zhuojia Xu, Chen Chen, Liguang Xu, Chuanlai Xu, Maozhong Sun and Hua Kuang**

Supporting information

METHODS

Chemicals

Hydrogen tetrachloroaurate trihydrate ($HAuCl_4 \cdot 3H_2O$), cetyltrimethylammonium bromide (CTAB, purity ≥ 99%), ascorbic acid (purity ≥ 99.9%), and ethylene glycol (EG, purity ≥ 99%) were obtained from Sigma-Aldrich, Shanghai, China. Hexadecyl trimethyl ammonium chloride (CTAC, purity ≥ 95.0%) was obtained from TCI Chemical Industries, Shanghai, China. Potassium iodide (KI, purity ≥ 99%) and sodium hydroxide (NaOH, purity ≥ 95%) were purchased from Sinopharm Chemical Reagent Co., Ltd, Shanghai, China. Cysteine-phenylalanine dipeptide (purity ≥ 98%), was purchased from Sangon Biotechnology, Shanghai, China. All aqueous solutions were prepared with deionized water (18.2 MΩ; Millipore). A 594 nm laser was purchased from Changchun New Industries Optoelectronics Technology Co., Ltd, Changchun, China. A quarter wave plate for 594 nm was purchased from Thorlabs. Map2 monoclonal antibody, GFAP polyclonal antibody, Alexa-Fluor®-488-conjugated goat anti-rabbit IgG (H+L), goat anti-mouse IgG (H+L), Alexa-Fluor®-Plus-555-conjugated SOX9 polyclonal antibody, the Fluo-4 Calcium Imaging Kit, anti-Aβ(1-42) polyclonal antibody, anti-p-tau monoclonal antibody, anti-BrdU monoclonal antibody, anti-DCX monoclonal antibody, and 4’6-diamidino-2-phenylindole (DAPI) were purchased from Thermo Fisher Scientific. The p-tau ELISA kit and beta amyloid ELISA kit were purchased from Thermo Fisher Scientific. The Cell Counting Kit-8 was purchased from Beyotime. Mouse neural

stem cell lines were obtained from the brains of self-cultured BALB/C fetal mice. The following were also obtained: neurobasal medium (Invitrogen/Gibco, cat. no. 21103-049), DMEM and Ham's F-12 medium at a 1:1 ratio (DMEM/F-12; Omega Scientific, cat. no. DM-25), B27 serum-free supplement (B27; Invitrogen/Gibco, cat. no. 17504-044), N2 supplement (N2; Invitrogen/Gibco, cat. no. 17502-408), GlutaMAX (Invitrogen/Gibco, cat. no. 35050-038), antibiotic-antimycotic (Anti-Anti; Invitrogen/Gibco, cat. no. 15240-062), basic fibroblast growth factor-2 (PeproTech, cat. no. 100-18B-B), epidermal growth factor (EGF; PeproTech, cat. no. 100-15), and Trypsin-EDTA (10×, Invitrogen/Gibco, cat. no. 15400-054).

Instrumentation

Circular dichroism spectra were obtained using Chirascan-Plus Circular dichroism. The measurement temperature was maintained at 25 °C. The scanning range for all samples was 300-1000 nm under high-purity nitrogen. The ultraviolet-visible (UV-Vis) spectra were obtained with a UNICO 2100 PC UV-Vis spectrophotometer and processed with OriginLab software. SEM (JEOL JSM-7401F) at an accelerating voltage of 2.0 kV, and Tecnai G2 F30 S-Twin field-emission transmission electron microscopy, operating at 200 KV, were carried out. Confocal images were obtained with a Leica LSM880 confocal fluorescence microscope (Leica Microsystems, Wetzlar, Germany). Histological sections were stained with H&E and photographed with a Leica optical microscope. The flow-cytometric assessment of NSCs markers with the analysis of mean fluorescent signals was performed on a BD

FACSAria™.

Experimental Section

Synthesis of chiral NPs

Au NPs were synthesized according to a previously reported method[1]. Firstly, the seed (trigonal prism) was synthesized. Then, 1.6 mL of 0.1M CTAC was added to 8 mL deionized water, followed by 75 μL of 10 mM KI. Sodium tetrachloroaurate solution was prepared by mixing $HAuCl_4$ and NaOH at a ratio of 1:1. Then, 100.4 μL was added to the reaction solution and set aside. 80 μL of 64 mM ascorbic acid was then added. Finally, 10 μL of 0.1M NaOH was quickly added, stirred vigorously for 30 s, and left to stand for 10 min. The prism was centrifuged at 6,200×g for 5 min, and then suspended in 500 μL of 1 mM CTAB.

CTAB (0.8 mL of 10 mM) and 0.2 mL of 10 mM $HAuCl_4$ were added to 3.95 mL deionized water to form the growth solution. Ten minutes later, 0.475 mL of 40 mM ascorbic acid was injected into the growth solution. Then, 5 μL of 4 mM L/D-cysteine-phenylalanine dipeptide and 50 μL seed solution were added to the growth solution, and thoroughly mixed. Chiral NPs with high *g*-factors were prepared by polarizing light (wavelength: 594 nm; strength: 84 mW/cm^2) and applied during growth. The reaction solution was injected into a quartz colorimetric dish and immediately irradiated with the corresponding polarized light for 30 min (L-type NPs or D-type NPs). L-type NPs were exposed to LCP light, and D-type NPs were exposed to RCP light. With regard to the nanoprism, no chiral molecules were

observed during growth. For further biological applications, the NPs (60 nM) were modified with 4 mM MPEG-SH (MW: 2000) for 4 h.

Cell lines and incubation conditions

Extraction of NSCs from the hippocampus: All *in vivo* experiments were approved by the Animal Welfare Committee of Jiangnan University (Wuxi, China). First, a fetal mouse (within 24 h of birth or shortly before birth) was euthanized. The mice were deeply anesthetized by intraperitoneal injection of pentobarbital sodium 230 mg/kg, and then euthanized by decapitation. Using small scissors, a medial caudal cut along the head from above the spinal cord area of the neck was made and the skin from the head was removed. Then a longitudinal incision at the base of the skull was made and continued along the sagittal line. With regard to the skull, one hemisphere at the bottom of the incision, was then clamped with a sharp end clamp and stripped outward to expose the brain. This was repeated in the other hemisphere. The brain tissue was removed for subsequent procedures and placed in an ice bath. The cerebellar tissue was removed with curved, pointed forceps, and the brain was bisected along the sagittal line, slowly stripping out the hippocampus. Then, some tissue cells in the hippocampus were picked out with elbow tweezers and placed in RPMI 1640 culture medium at 4 °C for later use. After all mouse brain tissues were extracted, they were collected together into a 15 mL sterile centrifuge tube, 2 mL trypsin was added, and the tissue samples were repeatedly blown with a pipettor for 2 min to disperse them as much as possible. They were then placed in a 37 °C water

bath and incubated for 15 min. Next, 4 mL 1640 medium containing 10% serum was added immediately after incubation to terminate trypsin digestion. The samples were blown with a pipettor for 2 min, centrifuged at 1300 ×g for 3 min, the precipitate was collected and then re-suspended in 2 mL brain tissue cell culture medium. The cells were placed in an incubator and cultured at 37 °C in a humidified atmosphere of 5% CO_2 for adherent growth.

Cell viability

NSCs (1.0×10^6) were inoculated in 96-well plates with 3 wells for each concentration of chiral NPs. The cells were cultured for 48 h at 37 °C and then the medium was replaced with 100 μL fresh OpTI-MEM (Life Technologies). Cells were treated with 10 μL Cell Counting Kit-8 (CCK-8, Beyotime) for 1 h, and the absorbance (A) of each well was measured at 450 nm wavelength using a microplate analyzer, and the relative cell viability (%) was calculated as (Atest/Acontrol) × 100.

Immunofluorescence staining

Differentiated cells were fixed with 4% paraformaldehyde for 15 min, permeabilized with 0.1% Triton X-100 for 3 min, and then blocked with 10% (v/v) bovine serum albumin in PBS for 2 h at room temperature or overnight 4° C. Cells were washed three times with PBS, and incubated with 1:500-diluted primary mouse anti-Map2 monoclonal antibody and rabbit anti-GFAP polyclonal antibody for 2 h. After incubation with primary antibodies, the cells were washed three times with PBS

(3 min) and then incubated for 2 h with 1:200-diluted secondary antibodies: conjugated 488-conjugated goat anti-rabbit IgG (H+L) and conjugated 555-conjugated Goat anti-mouse IgG (H+L). The cells were washed with PBS for 5 min, and the nuclei were counterstained with DAPI (0.25 μg/mL; Molecular Probes) for 12 min. Cells were observed under an inverted fluorescence microscope (Leica DMI8).

Flow cytometry

Co-staining with anti-Map2 monoclonal antibody and anti-GFAP polyclonal antibody was used to determine the proportions of differentiated neurons and astrocytes in different culture conditions. For flow cytometry analysis, cells were fixed, permeabilized, blocked, and incubated with primary antibodies diluted 1:500 for 2 h. Cells were finally incubated with 1:200-diluted secondary antibodies (Alexa-Fluor®-488-conjugated goat anti-rabbit IgG [H+L] and Alexa-Fluor™-Plus-555-conjugated goat anti-mouse IgG [H+L]) for 2 h. Flow cytometry was performed using the BD-type FACSAria, and FlowJo was used for data analysis.

Differentiation and identification of NSCs

To analyze the effects of assembly and CPL on neural cell differentiation, NSCs were placed in culture medium with chiral NPs under different light conditions. After 7 days of co-culture, the expression of Map2 and GFAP was observed with a confocal scanning microscope. DAPI was used to counterstain the nuclei blue.

Ca^{2+} imaging

To assess neural activity, cellular calcium dynamics in differentiated NSCs were monitored using the Fluo-4 Calcium Imaging Kit (Life Technologies). The differentiated NSCs were washed once with physiological buffered saline, and 2 mL of Fluo-4 AM loading solution (100 μL of 100× PowerLoad™ concentrate, 10 μL of 1000× Fluo-4 AM, 10 mL of physiological buffer) was added and incubated at 37 °C. After 15–30 min incubation with the Fluo-4 AM loading solution the cells were washed once with physiological buffer. After adding physiological buffer (2 mL), the cells were ready for live cell imaging. Dynamic changes in fluorescence intensity were measured by time-lapse imaging using a Leica LSM880 confocal fluorescence microscope at a rate of 30 ms/frame.

Global gene expression analysis

Total RNA per sample was extracted from NSCs using the TRIzol Reagent (Invitrogen)/RNeasy Mini Kit (Qiagen)/other kits. Total RNA from each sample was quantified and qualified by an Agilent 2100 Bioanalyzer (Agilent Technologies, Palo Alto, CA, USA), NanoDrop (Thermo Fisher Scientific) and 1% agarose gel. Approximately 1 μg of total RNA with a RNA integrity number value greater than 6.5 was used for subsequent library preparation. Next-generation sequencing library preparations were constructed according to the manufacturer's protocol. The poly(A) mRNA Magnetic Isolation Module or rRNA Removal Kit for poly(A) mRNA isolation was used. mRNA was fragmented and primed using First Strand Synthesis

Reaction Buffer and random primers. The first-strand cDNA was synthesized with ProtoScript II reverse transcriptase, and the second-strand cDNA was synthesized with a second-strand synthase mix. The purified double-stranded cDNA was then treated with End Prep Enzyme Mix to repair both ends, and then adapters were added at both ends by T-A ligation. Using beads to screen the size of adaptor-ligated DNA, a fragment of about 420 bp (insert length of about 300 bp) was obtained. Each sample was subjected to 13 cycles of PCR amplification with P5 and P7 primers, the sequences carried by both primers could be annealed to flow cytometry for bridging PCR, and the 6-base index carried by the P7 primer allowed multiplexing. PCR products were washed with beads, verified with Qsep100 (Bioptic, Taiwan, China), and quantified with a Qubit 3.0 fluorometer (Invitrogen, Carlsbad, CA, USA). Libraries with different metrics were then multiplexed and loaded onto an Illumina HiSeq instrument according to the manufacturer's instructions (Illumina, San Diego, CA, USA). Sequencing was performed in a 2x150 bp paired-end (PE) configuration; HiSeq Control Software (HCS) + OLB + GAPipeline-1.6 (Illumina) for image analysis and base calling on a HiSeq instrument. Sequences were processed and analyzed with GENEWIZ.

Stereological injection

Ten-month-old prp-hap/hPS1 double transgenic mice were used for *in vivo* experiments, and siRNA at a final concentration of 2 μg/mL was injected into the mouse brain. Each mouse was stereotaxically injected at a rate of 0.3 μL/min.

Injection coordinates relative to bregma anteroposterior, −1.7 mm; mediolateral, ± 1.5 mm; dorsoventral, −1.5 mm in the same location. Then 1 μM retinoic acid and chiral NPs (5 mg/kg) were mixed and injected again at the same site.

All *in vivo* experiments were selected for LP irradiation and approved by the Animal Welfare Committee of Jiangnan University.

Immunohistochemical and immunofluorescent staining of brain sections

Isolated brains were perfused and fixed with 4% PFA in PBS. Brains were placed in 4% paraformaldehyde in 0.1 M phosphate buffered saline overnight at 4°C, followed by 30% sucrose solution in 0.05 M PBS for 48 h at 4 °C. Coronal sections (30 μm thick) were prepared using a cryostat. After rinsing three times (5 min each time) in PBS, the sections were blocked with 3% normal donkey serum in PBST (PBS + 0.3% Triton X-100) for 2 h, and then mixed with primary antibody (1:10 000) for 24 hat 4 °C. After three washes (5 min each) in PBST, the sections were incubated with secondary antibody (1:500) for 2 h at room temperature. The sections were rinsed with PBST. Confocal fluorescence microscopy images were acquired under a 20× objective. The brain tissue, liver, kidney and other major organs were histopathologically observed using an H&E staining kit.

Morris water maze test and Nissl staining

The memory test in the AD mouse model was performed using the morris water maze (MWM) method. Before the water maze test, four trials per day were performed

for 5 consecutive days. The position of the platform remained unchanged throughout the experiment. Data were collected automatically using the Nikon tracking system. To acquire the data, we divided the swimming pool with imaging lines into four equal quadrants. At the beginning of each trial, a rat was placed at one of four fixed starting points and allowed to swim for 120 s or until it found the platform. The trial was terminated if the animal was unable to find the platform within 120 s. The proportion (%) of the animals' swimming time in the target quadrant to the total swimming time in the maze was recorded for statistical analysis.

After the MWM experiment, the mice were euthanized and their hearts were perfused with 60 mL of cold saline. The whole brain was fixed with 10% formalin, embedded in paraffin, and sectioned at 3 μm. Nissl-stained sections were used to detect Nissl bodies in the cytoplasm of hippocampal neurons.

Statistical Analysis

All the results were reported as a mean with standard deviation. The significance test method of the paper was Student's T-test method, and marked in the corresponding position. The software used was GraphPad Prism 6.01(GraphPad Software, USA), and OriginPro 2022 (OriginLab, USA). Sample size (n) of independent repeated experiments for each statistical analysis was given in the figure legends. Differences were considered significant at $p < 0.05$.

Supplementary Figures:

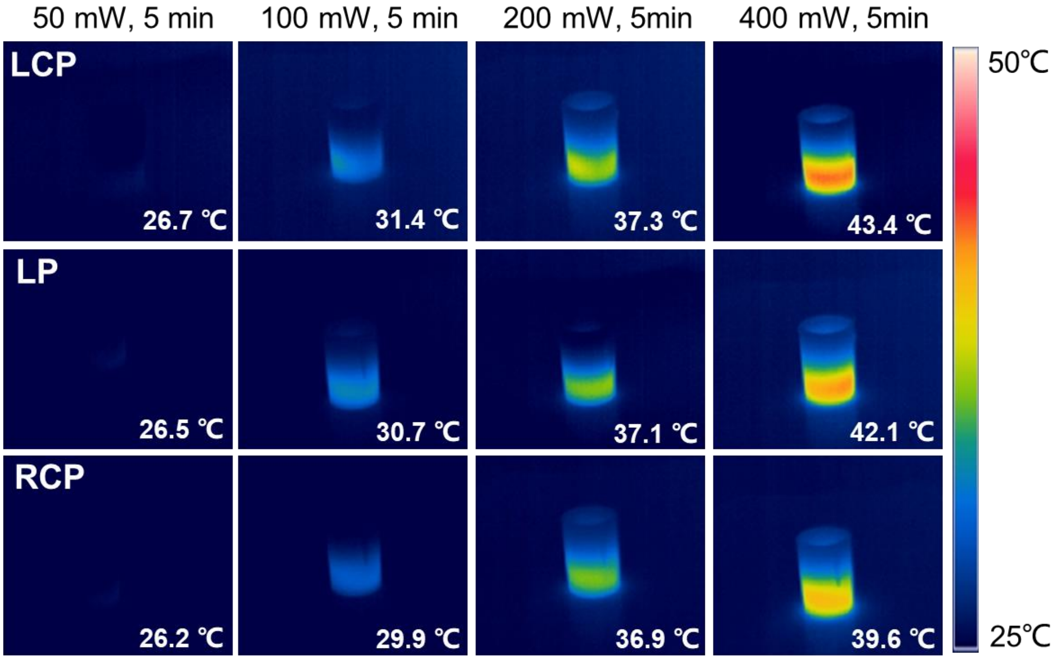

Figure S1. Thermal images of L-type NPs (200 μg/mL) under different NIR light irradiations (50, 100, 200 and 400 mW/cm^2) with CPL.

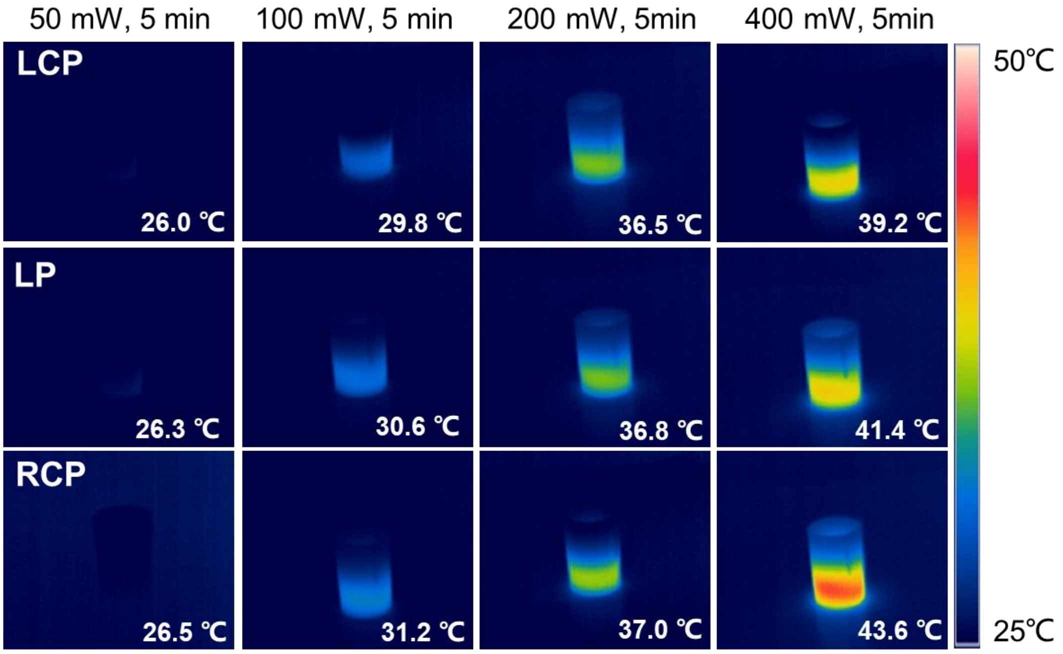

Figure S2. Thermal images of D-type NPs (200 μg/mL) under different NIR light irradiations (50, 100, 200 and 400 mW/cm^2) with CPL.

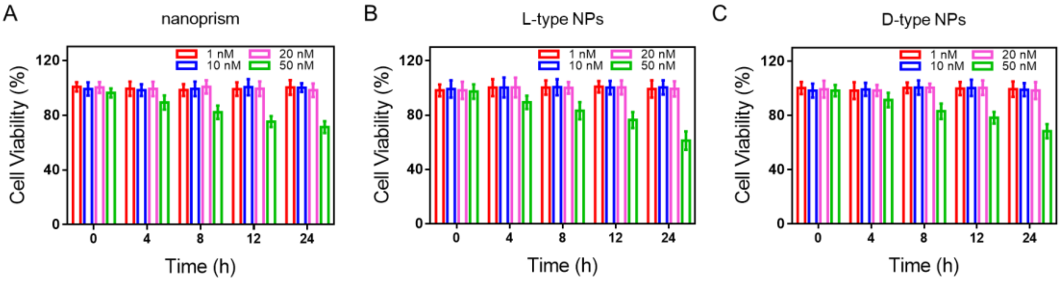

Figure S3. Cell Viability of NSCs incubated with nanoprism (**A**), L-type NPs (**B**) and D-type NPs (**C**) at different concentrations and times. Data are presented as mean ± s.d. (n = 5).

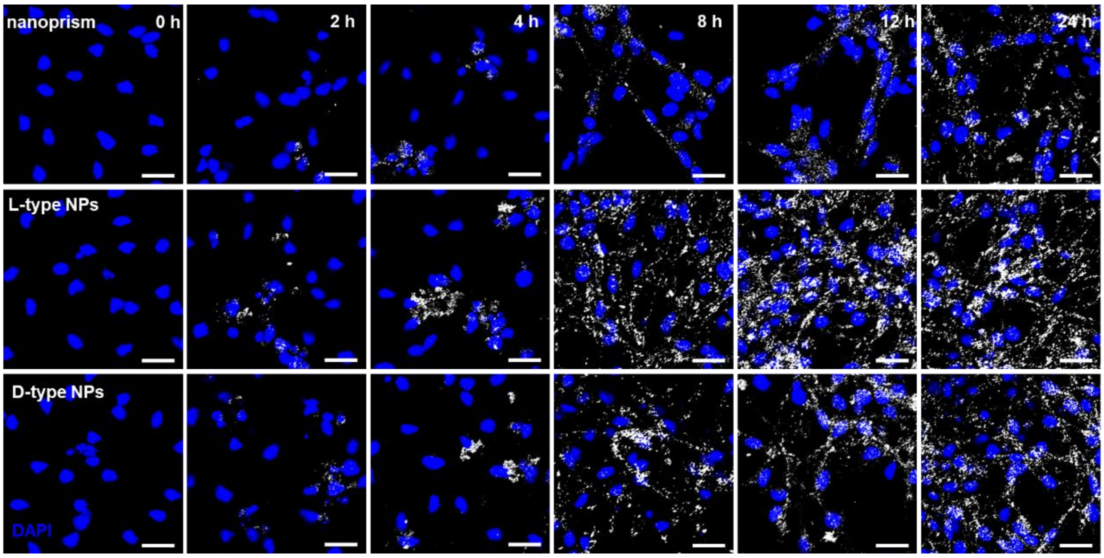

Figure S4. The NSCs incubated with chiral NPs (nanoprism, L-type NPs and D-type NPs) for different time. Blue: DAPI for the nuclei. Scale bar, 20 μm.

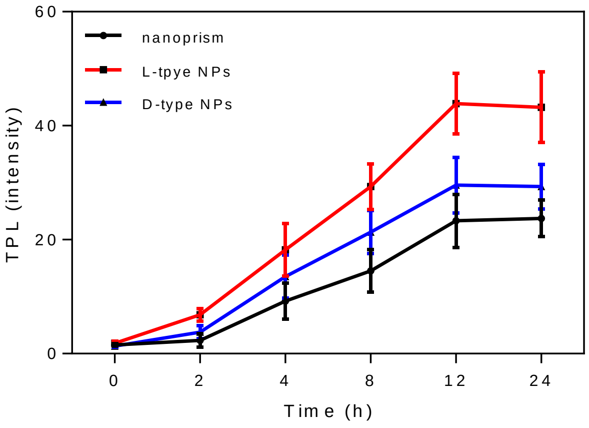

Figure S5. Gray values of chiral NPs (nanoprism, L-type NPs and D-type NPs)

incubated in NSCs for different time.

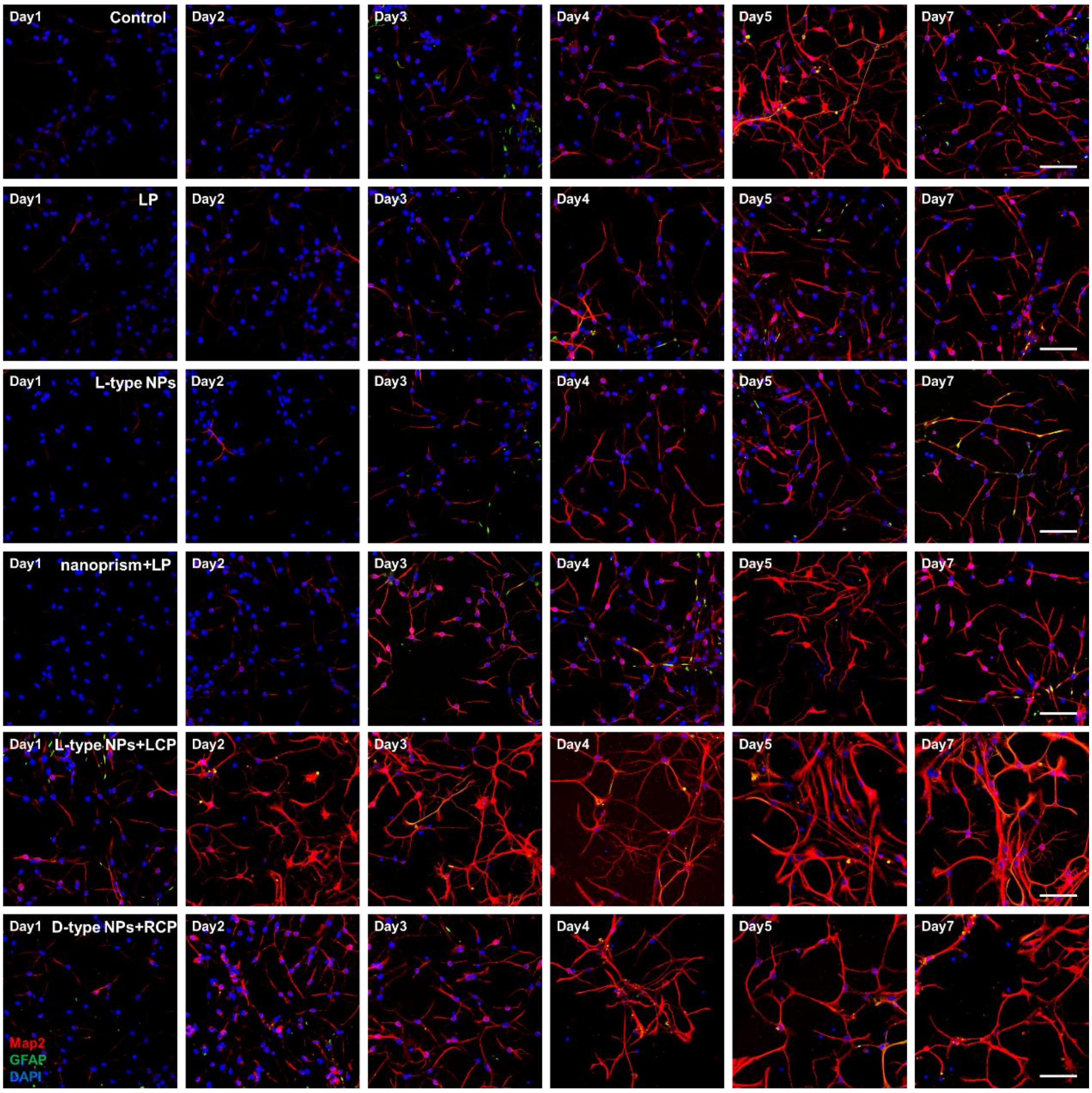

Figure S6. Confocal images of NSCs incubated for 7 days under different experimental conditions (Control, LP light only, L-type NPs only, nanoprism under LP light, L-type NPs under LCP light and D-type NPs under RCP light). In the group containing the material, the material was incubated with NSC for 12h every day, the medium was replaced and then irradiated with CPL (200 mW/cm^2, 5min), incubated for another 12h, and the new medium and material were replaced. Cells without nanoparticles or light exposure were used as control. Red: Map2 for mature neurons. Blue: DAPI for the nucleus. Green: GFAP for astrocytes. Scale bar, 100 μm.

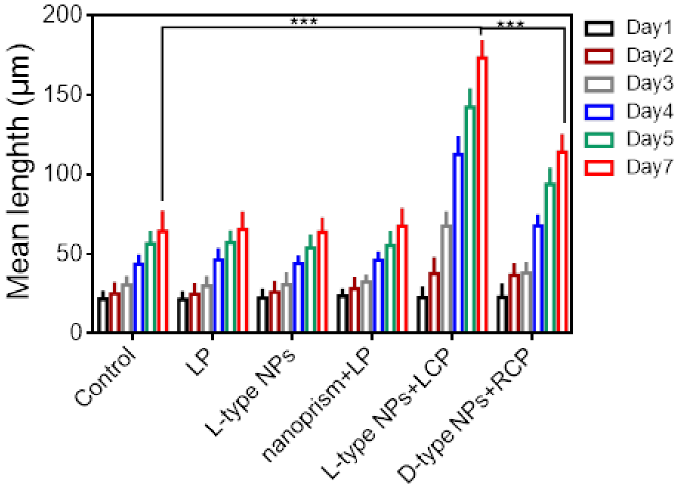

Figure S7. Mean lengths of neurites in differentiated NSCs after incubated with different experimental conditions. ***p < 0.001. Data are presented as the mean ± s.d. (n=5).

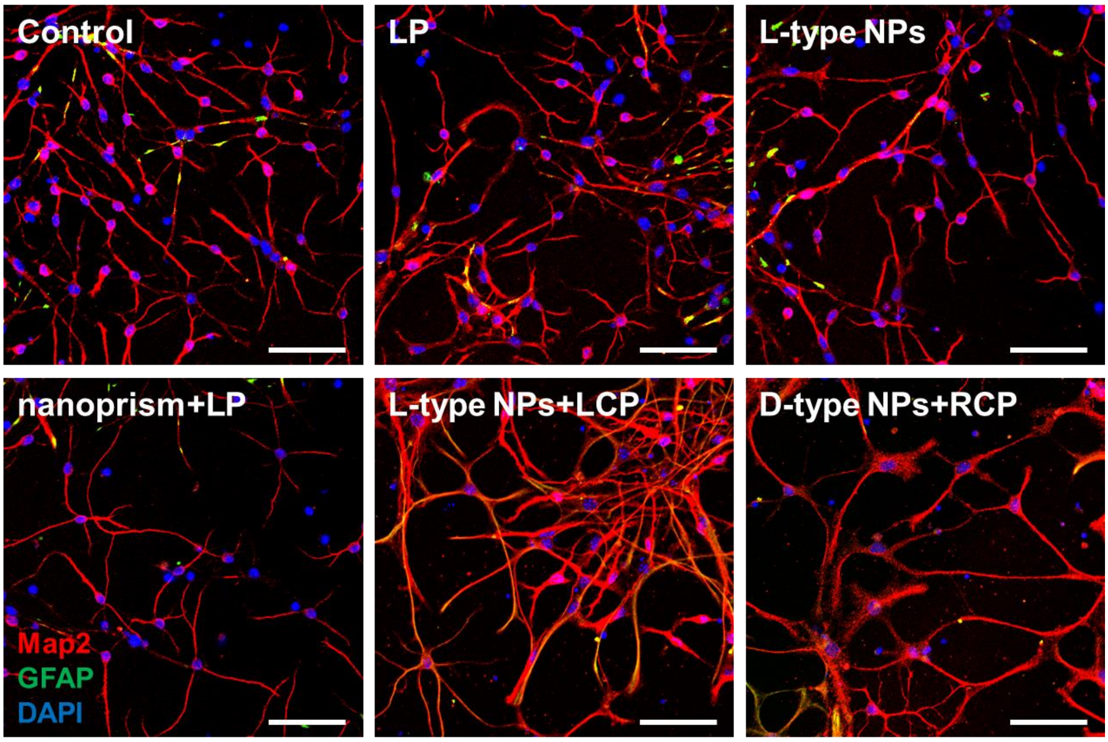

Figure S8. Confocal images of NSCs incubated with golden triangle nanoparticles with different g-factor values under the same conditions of the material entering the cell, and subsequently illuminated with CPL (200 mW, 5min) for 7 days. Red: Map2 for mature neurons. Blue: DAPI for the nucleus. Green: GFAP for astrocytes. Scale bar, 100 μm.

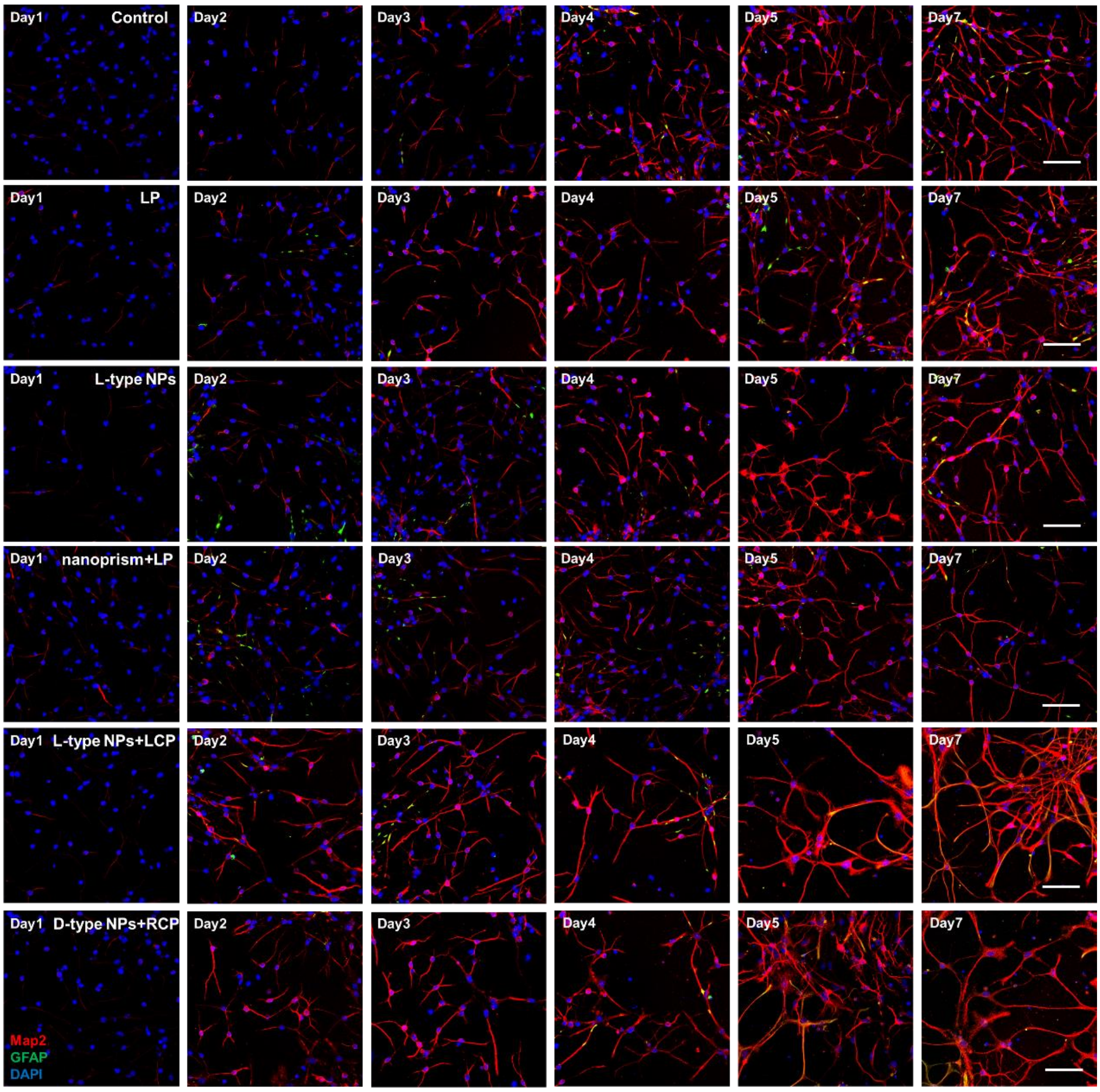

Figure S9. Confocal images of NSCs incubated with different experimental conditions (Control, LP light only, L-type NPs only, nanoprism under LP light, L-type NPs under LCP light and D-type NPs under RCP light) under the same concentration of the material entering the cells. In the group containing the material, the material was incubated with NSC for 12h every day, the medium was replaced and then irradiated with CPL (200 mW/cm^2, 5min), incubated for another 12h, and the new medium and material were replaced. Cells without nanoparticles or light exposure were used as control. Red: Map2 for mature neurons. Blue: DAPI for the nucleus. Green: GFAP for astrocytes. Scale bar, 100 μm.

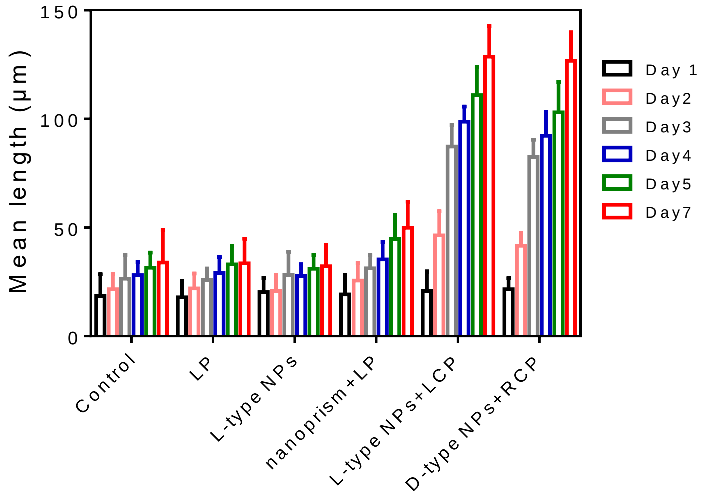

Figure S10. Mean lengths of neurites in differentiated NSCs incubated with different experimental conditions under the same concentration of the material entering the cells. Data are presented as the mean ± s.d. (n=5).

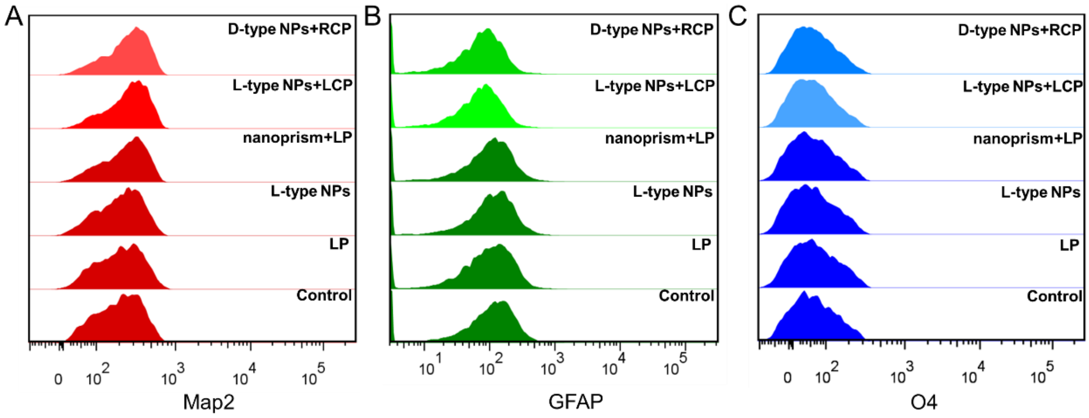

Figure S11. The differentiated proportion of astrocyte, oligodendrocyte, and neuron after NSCs cultured with different experimental conditions under the same concentration of the material entering the cells by flow cytometry.

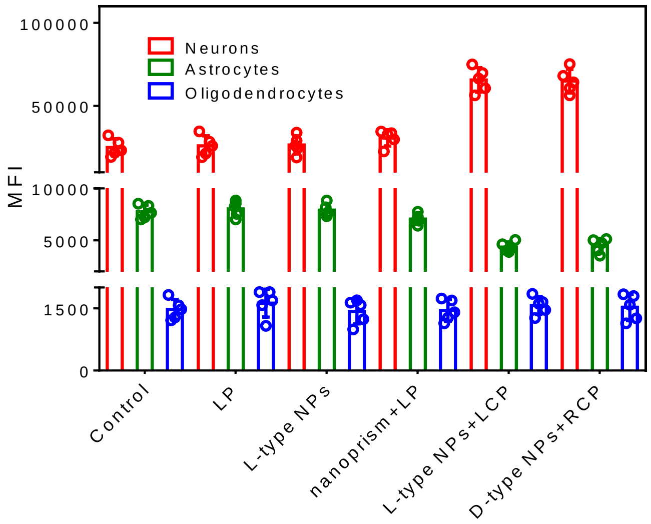

Figure S12. Mean fluorescence intensity (MFI) in **Fig. S12**. Data are presented as the mean ± s.d. (n=5).

The effect of chiral NPs in the same intracellular concentration on the proliferation and differentiation of NSCs was also verified. On the premise that the intracellular chiral NPs concentration was consistent, the medium and materials were changed every day, and the cells were cultured under CPL irradiation for 7 days (200 mW/cm^2, 5 min/day), and the neurite length of NSCs was observed by confocal microscope. The results indicated that chiral NPs could promote the neurite length under the CPL illumination (**Figures S8-10**). Among them, L-type NPs group and D-type NPs group changed significantly under CPL light illumination. After seven days of differentiation, the neurite length of NSCs in L-type NPs group was 128.64 μm, while that of D-type NPs group was 126.72 μm, increased by 3.79 and 3.73 times compared with the control group. The change of cell differentiation ratio was also verified by flow cytometry, and it was found that chiral NPs could promote the differentiation of NSCs towards neurons under the CPL illumination (**Figures S11-**

12). Under LCP illumination, the proportion of neurons in L-type NPs group increased by 1.263 times compared with the control group. Under RCP illumination, D-type NPs group increased by 1.260 times. These results showed that chiral NPs could improve the proportion of NSCs differentiation into neurons under the CPL illumination.

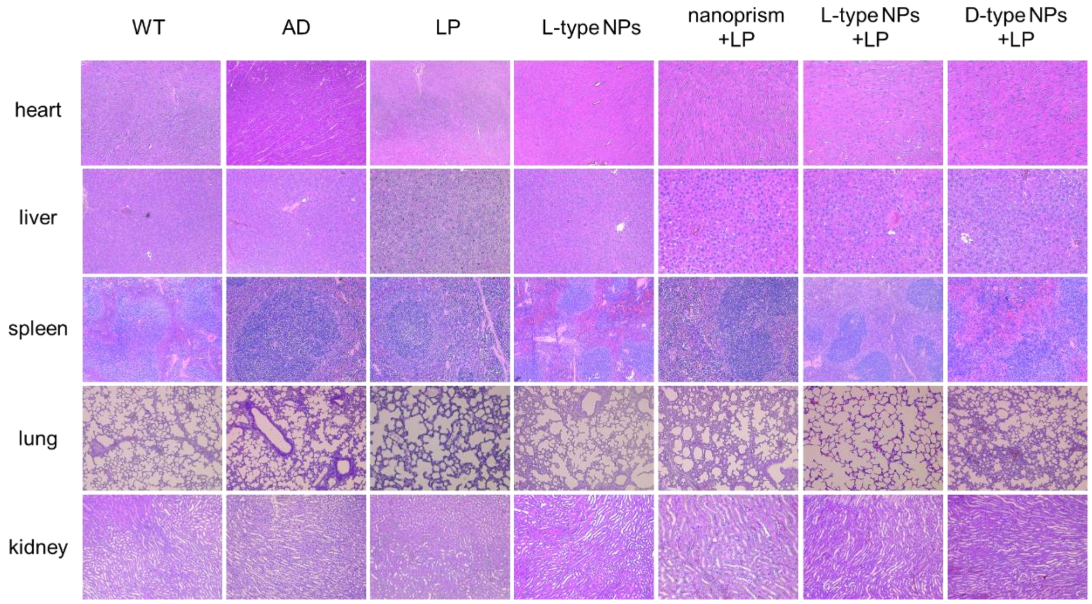

Figure S13. Hematoxylin and Eosin (H&E) stained different organs tissue (heart, liver, spleen, lung ang kidney) after different treatments (Normal saline injection was used as control group; LP irradiation group only; L-type NPs only; nanoprism, L-type NPs or D-type NPs under LP irradiation. In the added LP light group, the light was 12 hours a day, intensity 600 mW/cm^2). Scale bar, 100 μm. (n = 5).

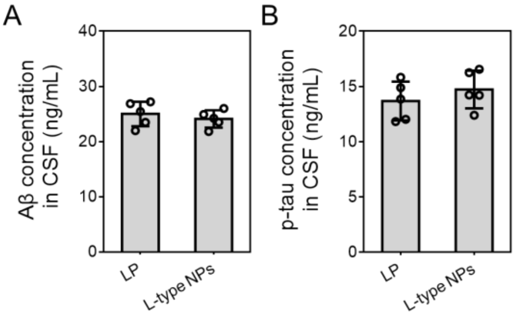

Figure S14. Aβ (**A**) and p-tau (**B**) concentration in cerebrospinal fluid of AD mice after different treatments. Data are presented as the mean ± s.d. (n=5).

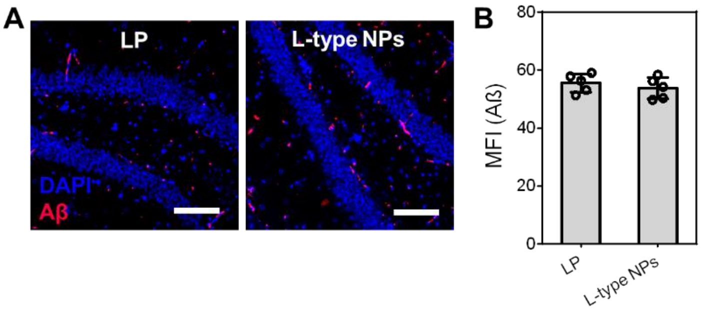

Figure S15. (**A**)Immunofluorescence of Aβ in the hippocampal of AD mice after different treatments. Scale bars, 50 μm. (**B**) Mean fluorescence intensity of Aβ in the hippocampal after different treatments. Data are presented as the mean ± s.d. (n=5).

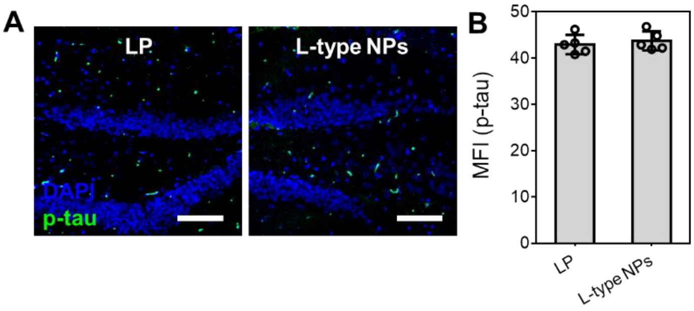

Figure S16. (**A**)Immunofluorescence of p-tau in the hippocampal of AD mice after different treatments. Scale bars, 50 μm. (**B**) Mean fluorescence intensity of p-tau in the hippocampal after different treatments. Data are presented as the mean ± s.d. (n=5).

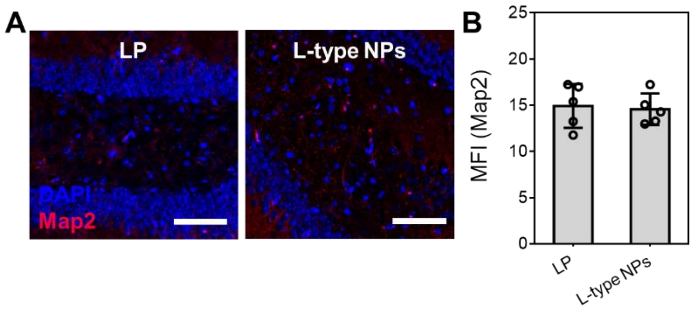

Figure S17. (**A**)Immunofluorescence of Map2 in the hippocampal of AD mice after different treatments. Scale bars, 50 μm. (**B**) Mean fluorescence intensity of Map2 in the hippocampal after different treatments. Data are presented as the mean ± s.d. (n=5).

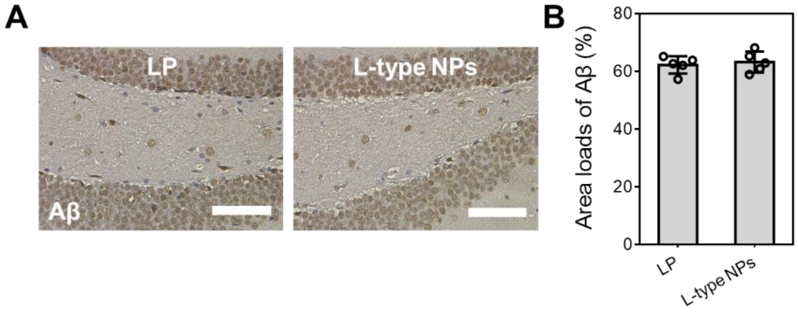

Figure S18. (**A**) Representative immunostaining of hippocampal sections for Aβ protein aggregates of AD mice after different treatments. Scale bars, 50 μm. (**B**) Quantitative analysis of Aβ **(A)** loads in the brains (hippocampus) after different treatments. Data are presented as the mean ± s.d. (n=5).

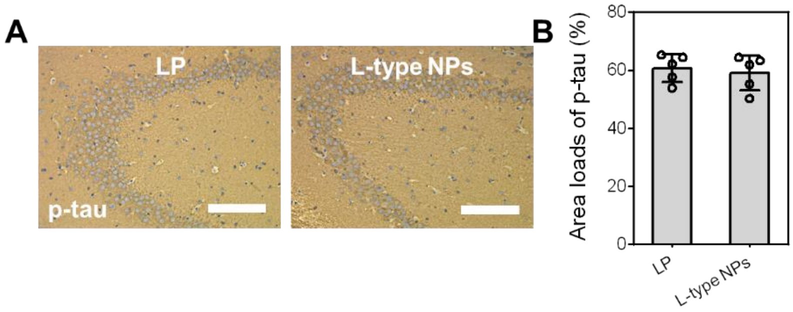

Figure S19. (**A**) Representative immunostaining of hippocampal sections for p-tau protein aggregates of AD mice after different treatments. Scale bars, 50 μm. (**B**) Quantitative analysis of p-tau **(A)** loads in the brains (hippocampus) after different

treatments. Data are presented as the mean ± s.d. (n=5).

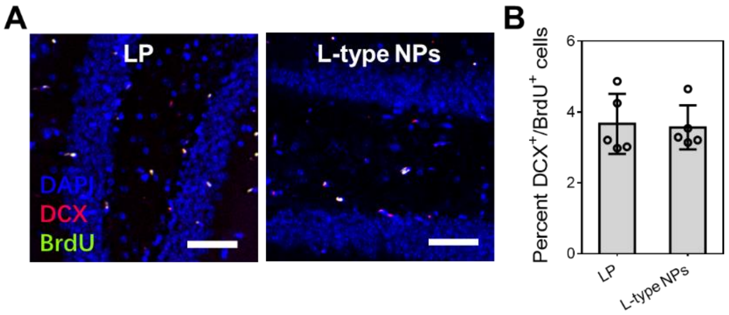

Figure S20. (**A**) Representative hippocampal section images of AD mice with different treatments, immunostained for DCX (red) to label immature neurons, BrdU (green) to label dividing cells, and DAPI (blue) to stain nuclei. Scale bars, 50 μm. (**B**) Quantification of the overall fraction of newborn hippocampal cells in **(A)** that underwent neuronal differentiation using stereological estimation. Data are presented as the mean ± s.d. (n=5).

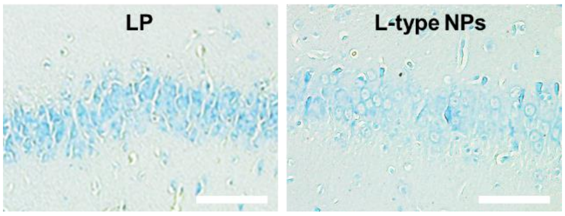

Figure S21. Nissl staining of neuro cells in the brains (hippocampus) of AD mice with different treatments.

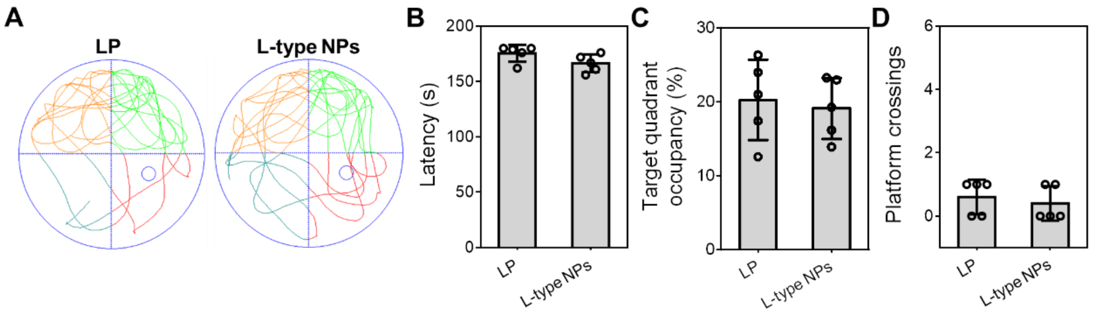

Figure S22. **(A)** The track sheets of AD mice after different treatments. (**B**) The latent period in water maze of AD mice to find the target quadrant after different treatments. (**C**) The time in the target quadrant of AD mice after different treatments. (**D**) The crosses times in target quadrant of AD mice after different treatments (*n* = 5

mice per group). Data are presented as the mean ± s.d. (n=5).

1. Xu, L.; Wang, X.; Wang, W.; Sun, M.; Choi, W. J.; Kim, J.-Y.; Hao, C.; Li, S.; Qu, A.; Lu, M.; Wu, X.; Colombari, F. M.; Gomes, W. R.; Blanco, A. L.; de Moura, A. F.; Guo, X.; Kuang, H.; Kotov, N. A.; Xu, C., Enantiomer-dependent immunological response to chiral nanoparticles. Nature 2022, 601 (7893), 366-373.
